# Bacteria, yeasts, and fungi associated with larval food of Brazilian native stingless bees

**DOI:** 10.1038/s41598-023-32298-w

**Published:** 2023-03-29

**Authors:** Ana Carolina Costa Santos, Luiza Diniz Ferreira Borges, Nina Dias Coelho Rocha, Vasco Ariston de Carvalho Azevedo, Ana Maria Bonetti, Anderson Rodrigues dos Santos, Gabriel da Rocha Fernandes, Raquel Cristina Cavalcanti Dantas, Carlos Ueira-Vieira

**Affiliations:** 1 Laboratory of Genetics, Institute of Biotechnology, Federal University of Uberlândia, Uberlândia, Brazil; 2 Department of Molecular Medicine, University of Rome La Sapienza, Rome, Italy; 3 Laboratory of Molecular and Cellular Genetics, Institute of Biological Sciences, Federal University of Minas Gerais, Belo Horizonte, Brazil; 4 Faculty of Computer Science, Federal University of Uberlândia, Uberlândia, Brazil; 5 Oswaldo Cruz Foundation, René Rachou Research Center, Belo Horizonte, MG Brazil

**Keywords:** Bacteria, Fungi, Bacterial genes, Fungal genes, Ecological genetics, Genetics, Microbial genetics, Sequencing

## Abstract

Stingless bees are a diverse group with a relevant role in pollinating native species. Its diet is rich in carbohydrates and proteins, by collecting pollen and nectar supplies the development of its offspring. Fermentation of these products is associated with microorganisms in the colony. However, the composition of microorganisms that comprise this microbiome and its fundamental role in colony development is still unclear. To characterize the colonizing microorganisms of larval food in the brood cells of stingless bees *Frieseomelitta varia*, *Melipona quadrifasciata*, *Melipona scutellaris*, and *Tetragonisca angustula*, we have utilized molecular and culture-based techniques. Bacteria of the phyla Firmicutes, Proteobacteria, Actinobacteria, and fungi of the phyla Ascomycota, Basidiomycota, Mucoromycota, and Mortierellomycota were found. Diversity analysis showed that *F. varia* had a greater diversity of bacteria in its microbiota, and *T. angustula* had a greater diversity of fungi. The isolation technique allowed the identification of 189 bacteria and 75 fungi. In summary, this research showed bacteria and fungi associated with the species *F. varia*, *M. quadrifasciata*, *M. scutellaris*, and *T. angustula*, which may play an essential role in the survival of these organisms. Besides that, a biobank with bacteria and fungus isolates from LF of Brazilian stingless bees was created, which can be used for different studies and the prospection of biotechnology compounds.

## Introduction

Stingless bees (Apidae, Meliponini) represent the most diverse eusocial bees, with more than 550 species distributed in tropical and subtropical regions^1–3^. Stingless bees are economically explored in Brazil's north and northeast regions due to the production of honey, wax, geopropolis, pollen, and the commercialization of colonies. However, the main socio-economic contribution is the pollination of native species^4–8^.

Brazilian stingless bee species present differences in behavior and caste differentiation; for instance, some stingless bees like *Frieseomelitta varia* and *Tetragonisca angustula* produce a royal cell to which the nurser worker will add a large quantity of LF (there is no different food such as royal jelly present in honeybee). Specifically, in the *Melipona* genus, there is no construction of royal cells and no production of exceptional food for the queen. The brood cells have the same size (volume) and receive the same quantity of equal food. *Melipona* has the “two loci/two alleles model” for cast differentiation, with queen development up to 25% under ideal food supply conditions^9,10^.

Despite the diverse mores of caste differentiation among stingless bee species, this process is regulated by juvenile hormone (JH) titter^9,11^. Although this hormone is produced by the *corpora allata* glands of stingless bees, the JH precursors can be obtained from metabolites of microorganisms associated with stingless bees. Recently, interesting works showed that *Scaptotrigona depilis* stingless bees have a mutualist relationship with fungi of the genera *Monascus* and *Zygosaccharomyces* present in larval food (LF)*,* influencing the development of these bees^12–14^. Besides those relationships well established, the frequency with C*andida apicola* and *Starmerella meliponinorum* yeasts have been found in pollen and honey of *Melipona quadrifasciata,* and *Tetragonisca angustula* suggests a mutualistic relationship between them^15^.

These bees are the primary pollinators in Brazil, and the hive structure is entirely different from other social bees. Despite the significant importance of stingless bees for pollination, little is known about the microbiological community inside the hive^16,17^. Nectar and pollen collected by foragers are stored in sealed cerumen pots, fermented by microorganisms to supply the necessary nutrients for the development of larvae and the survival of adults^17^. Through these processes, the nectar—transformed into honey by bees and fermented by microorganisms—will be the primary source of carbohydrates for these bees. In addition, fermented pollen is the source of proteins, lipids, and other nutrients^18–21^. LF results from a mix of fermented pollen, honey, and glandular secretions of nurse bees and is richly associated with microbial communities^16,22–25^.

Microorganisms in the hive contribute to the development of the bee's immune system, aid in food digestion, and defend the hives against pathogens^26–28^. Bacteria of the genera *Bacillus* and *Streptomyces* were found in beehives of *Melipona* and *Trigona*. These organisms produce antimicrobial substances against fungi and *Paenibacillus larvae* besides presenting fermentative potential^29–32^.

However, they present the structure of cerumen pots to fermented stock food in the colony (pollen and honey pots) and the production of LF^33^. Despite the recent advances in microbiota research associated with stingless bee colonies, the composition of the LF microbiome of these bees still needs to be determined. The role of the microorganisms in the feeding and the manutention of bee colonies stays an open field for investigation.

Due to the importance of microorganisms to stingless bees, knowing the microbial community present in beehives becomes necessary. In this work, to better understand and create a microorganism collection from stingless bees, we describe the microbiome present in LF of four species of Brazilian stingless (*Frieseomelitta varia*, *Melipona quadrifasciata*, *Melipona scutellaris*, and *Tetragonisca angustula)* bee reared in the urban area of the Cerrado (Brazilian Savanna) biome (one of the most important and large biomes in Brazil despite little-studied^34,35^). The LF microbial—cultivated and non-cultivated—communities were molecularly characterized by Next Generation DNA Sequencing (NGS) of rRNA regions—V3/V4 of 16S and ITS1—and characterized by culturing-based techniques. Besides that, we created a biobank with 189 bacteria and 75 fungus isolates from the LF of Brazilian stingless bees, which can be used for different studies and the prospection of biotechnology compounds.

## Results

### Raw and filtered data from NGS

A total of 1,002,306 amplicons with good quality were obtained after filtering, merged (F and R), and chimera removing from 1,315,720 raw reads of the region V3/V4 of 16S rRNA gene from microorganism present in LF of the stingless bees. Reads were clustered into 129 ASVs for the bacterial community. For the ITS1 region, a total of 624,309 amplicons with good quality were obtained after filtering, merged, and chimera removing from 4,420,973 raw reads. The reads were clustered into 300 ASVs fungal communities (Table 1).

**Table 1:** Reads processed by Dada2—R package.

	Input	Filtered	Merged	After chimera removed	ASVs
**16S data**					
*F. varia*	806,951	680,900	677,477	658,592	65
*M. quadrifasciata*	243,622	195,027	194,871	188,905	24
*M. scutellaris*	139,458	118,440	117,671	87,405	25
*T. angustula*	125,239	105,946	104,846	67,404	17
Total	1,315,270	1E + 06	1,094,865	1,002,306	129
**ITS data**					
*F. varia*	106,996	62,637	54,823	54,823	46
*M. quadrifasciata*	304,463	255,739	243,181	228,696	80
*M. scutellaris*	178,378	134,593	125,069	124,451	49
*T. angustula*	3,831,136	300,544	240,799	216,339	142
Total	4,420,973	753,513	663,872	624,309	300

### Bacterial community sequencing

**Figure 1:**
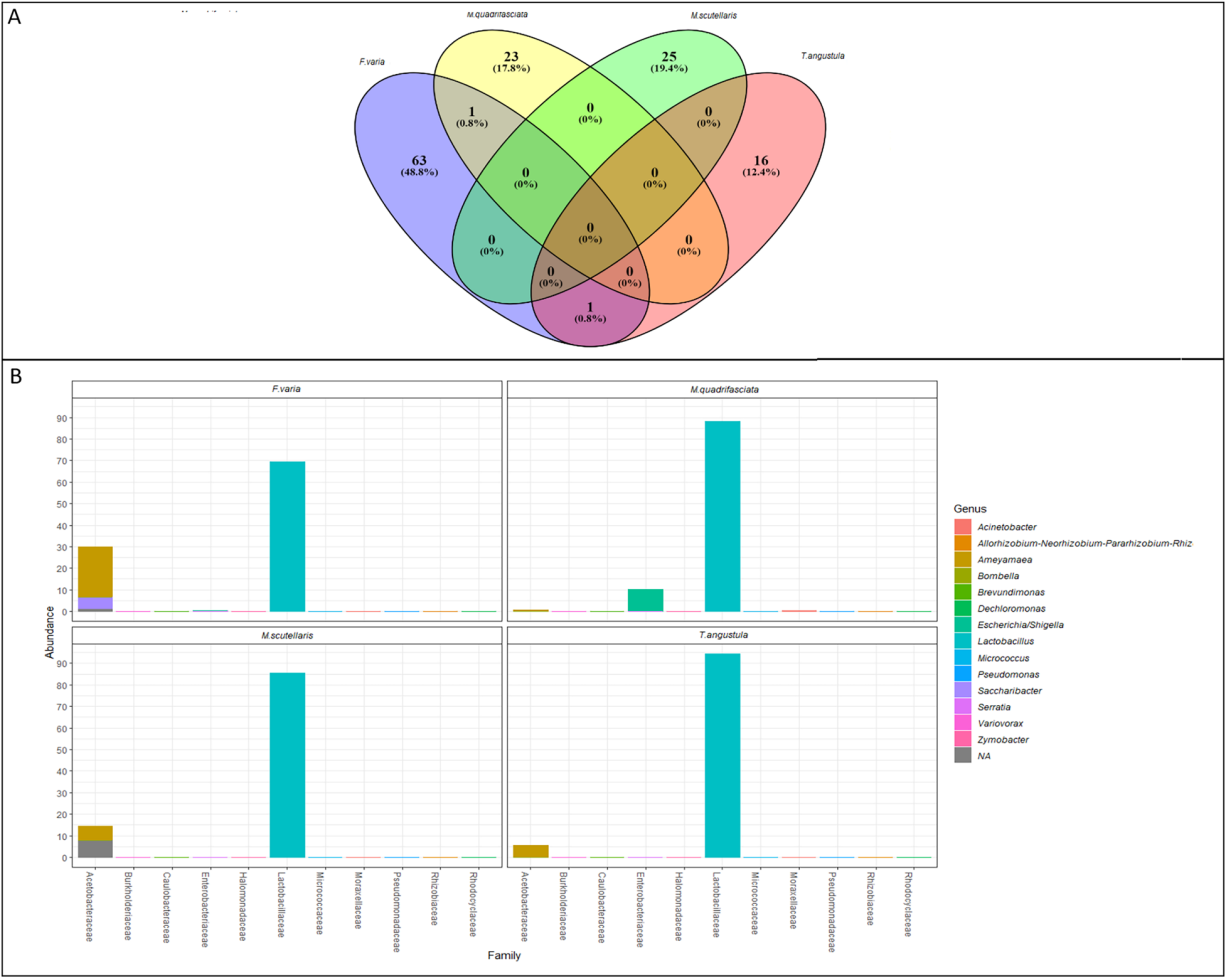
(**A**) Venn Diagram of ASVs classified for stingless bees; (**B**) Genus × Family histogram of 15 genera most abundant.

The bacterial composition analysis classified one hundred and twenty-nine ASVs (See Supplementary Table S1). No ASVs were found simultaneously in the LF of the four bee species. Sixty-three exclusive ASVs were detected in *F. varia* LF, 23 in *M. quadrifasciata*, 25 in *M. scutellaris*, and 16 in *T. angustula* (Fig. 1A).

The most abundant phyla observed in the LF of the four bee species were Firmicutes and Proteobacteria. The phylum Actinobacteria was not represented in the species *M. scutellaris* and *T. angustula* (Table 2). The ASVs were divided into 26 genera; the most abundant genus in all bees was *Lactobacillus* (Fig. 1B)*.* Fifteen exclusive bacteria genera showed in *F. varia*, five in *M. quadrifasciata*, and only the genus *Anthococcus* in *M. scutellaris* (See Supplementary Table S1)*.* No exclusive genus was found in the LF of *T. angustula*; the genus *Micrococcus* is expected in the LF of *F. varia* and *M. quadrifasciata*. Only five ASVs were classified at a species level, including *Bombella intestine, Corynebacterium lipophiloflavum*, *Serratia symbiotica* from LF of *F. varia*, and *Acinetobacter junii* and *Variovorax paradoxes* from LF of *M. quadrifasciata*.

**Table 2:** Abundance of bacterial phyla observed in *F. varia*, *M. quadrifasciata*, *M. scutellaris*, and *T. angustula* larval food.

Stingless bees	Actinobacteria	Firmicutes	Proteobacteria
*F. varia*	4	35	26
*M. quadrifasciata*	2	13	9
*M. scutellaris*	–	20	5
*T. angustula*	–	15	2
Total ASVs	6	83	42

### Fungal community sequencing

**Figure 2:**
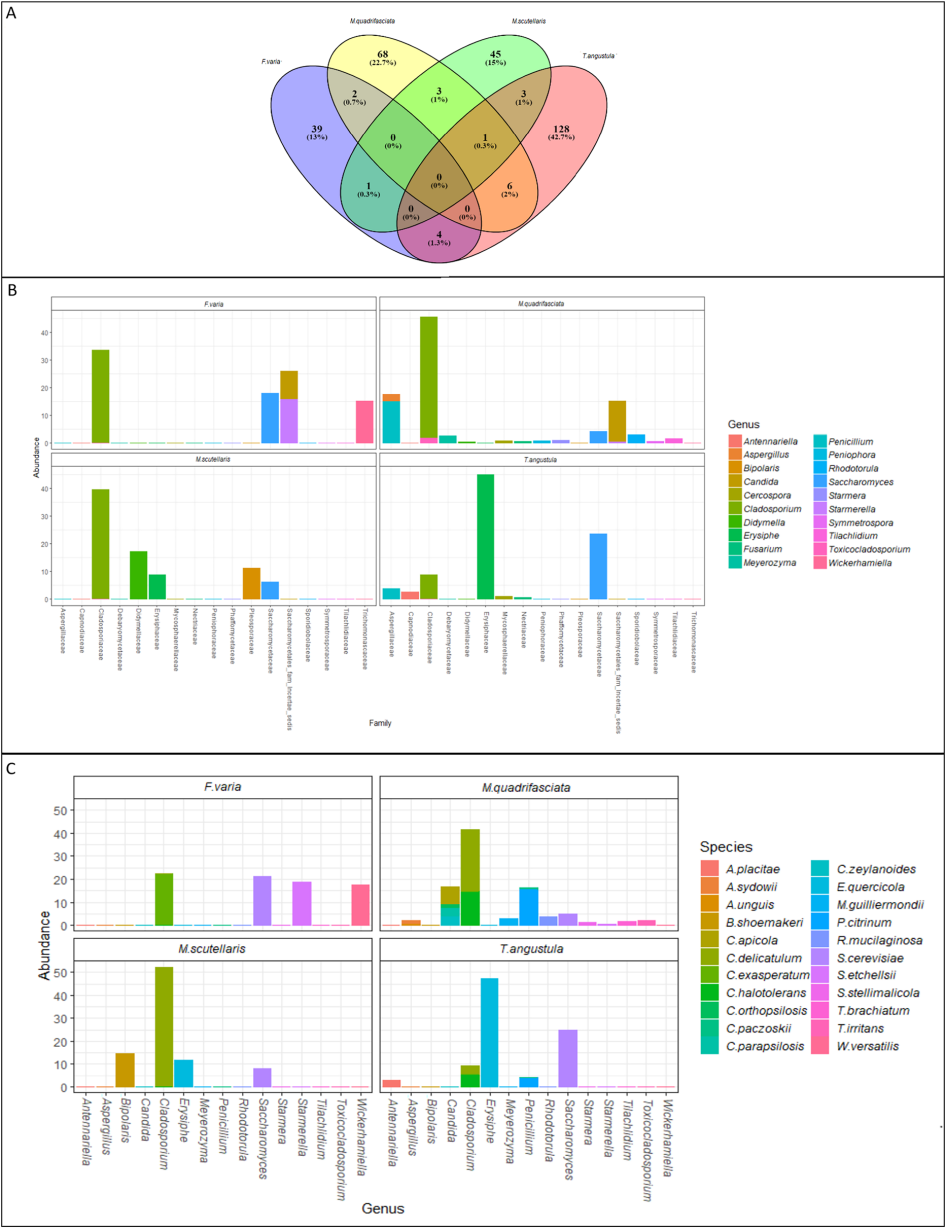
(**A**) Venn Diagram of ASVs classified for stingless bees; (**B**) Genus × Family histogram of 20 genera most abundant; (**C**) Species × Genera histogram of 22 species most abundant.

Three hundred ASVs were classified in fungal composition analysis from LF (See Supplementary Table S2). *F. varia* had 39 exclusive ASVs, *M. quadrifasciata* 68, *M. scutellaris* 45 and *T. angustula* 128. However, no common ASVs were found for the four stingless bee species (Fig. 2A).

Fungi of the phyla Ascomycota, Basidiomycota, Mucoromycota, and Mortierellomycota were identified. The phyla Ascomycota and Basidiomycota were the most abundant in LF of stingless bee species of this work. The only phylum in LF for the four species of stingless bees was Ascomycota (Table 3).

**Table 3:** Abundance of fungal phyla observed in *F. varia*, *M. quadrifasciata*, *M. scutellaris*, and *T. angustula* larval food.

Stingless bees	Ascomycota	Basidiomycota	Mortierellomycota	Mucoromycota
*F. varia*	11	–	–	–
*M. quadrifasciata*	57	12	1	1
*M. scutellaris*	14	3	–	–
*T. angustula*	30	1	–	1
Total ASVs	112	16	1	2

Stingless bees of the genus *Melipona* shared three exclusive ASVs, with ASV2 classified at the fungi kingdom, ASV44 as the genus *Didymella,* and ASV23 as *Saccharomyces cerevisiae*. The family emphasizing *M. quadrifasciata* and *F. varia* were Claridosporiaceae, and for *T. angutula,* Erysiphaceae (Fig. 2B) stood out. Forty-eight genera of fungi were identified, 11 were yeast genera, and 37 were filamentous fungi. In *F. varia* was found six genera of fungi, four species of yeasts, and two species of filamentous fungi. Different from the species *M. quadrifasciata*, *M. scutellaris,* and *T. angustula,* where filamentous fungal genera were most representative.

Fifty-eight fungal species were identified. The most abundant species in LF of *F. varia* were *Cladosporium exasperatum* and *Saccharomyces cerevisiae*. In *M. quadrifasciata* were *Cladosporium delicatulum* and *Penicillium citrinum*. In *M. scutellaris, Cladosporium delicatulum, Bipolaris shoemakeri* and *T. angutula, Erysiphe quercicola* and *Saccharomyces cerevisiae* (Fig. 2C). The *F. varia* LF showed three exclusive species, *M. quadrifasciata,* 21, *M. scutellaris,* five and *T. angustula,* 11.

## Richness and diversity indexes

**Figure 3:**
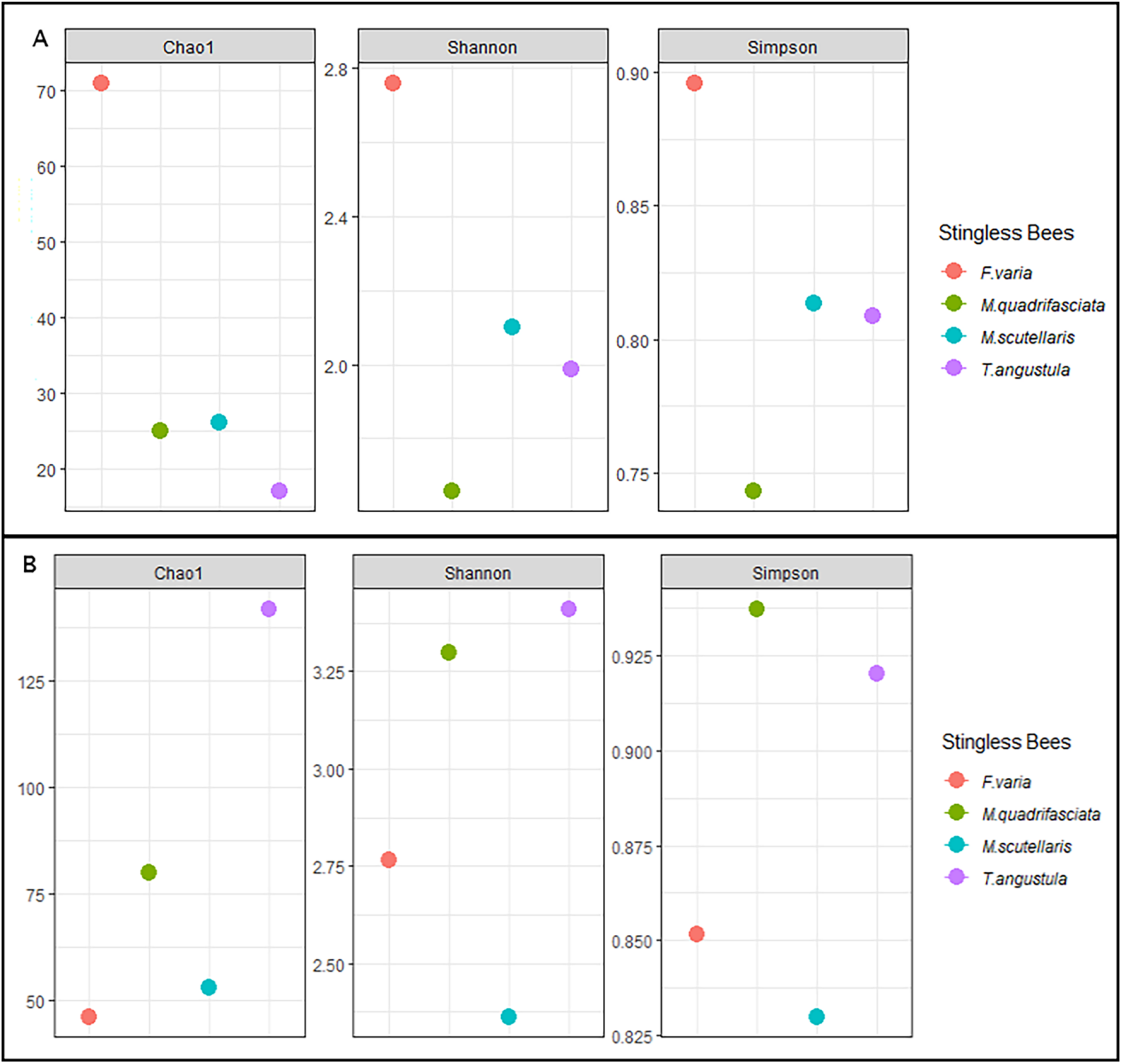
Alpha diversity index generated from the data of Bacterial (**A**) and Fungal (**B**) community sequencing.

The richness and diversity indexes from bacterial community sequencing were higher in the samples of *F. varia* LF regarding the number of bacterial species. The LF of *T. angustula* showed the lowest diversity, while *M. quadrifasciata* presented the lowest species diversity indexes. *Melipona scutellaris* and *T. angustula* showed a similar Shannon and Simpson diversity index (Fig. 3A).

For Fungal Community Sequencing, the higher richness of fungi species was found in *T. angustula* and the lowest in *F. varia*. *M. quadrifasciata* and *T. angustula* LF had the highest Simpson and Shannon indices, and *M. scutellaris* had the lowest indices (Fig. 3B).

**Figure 4:**
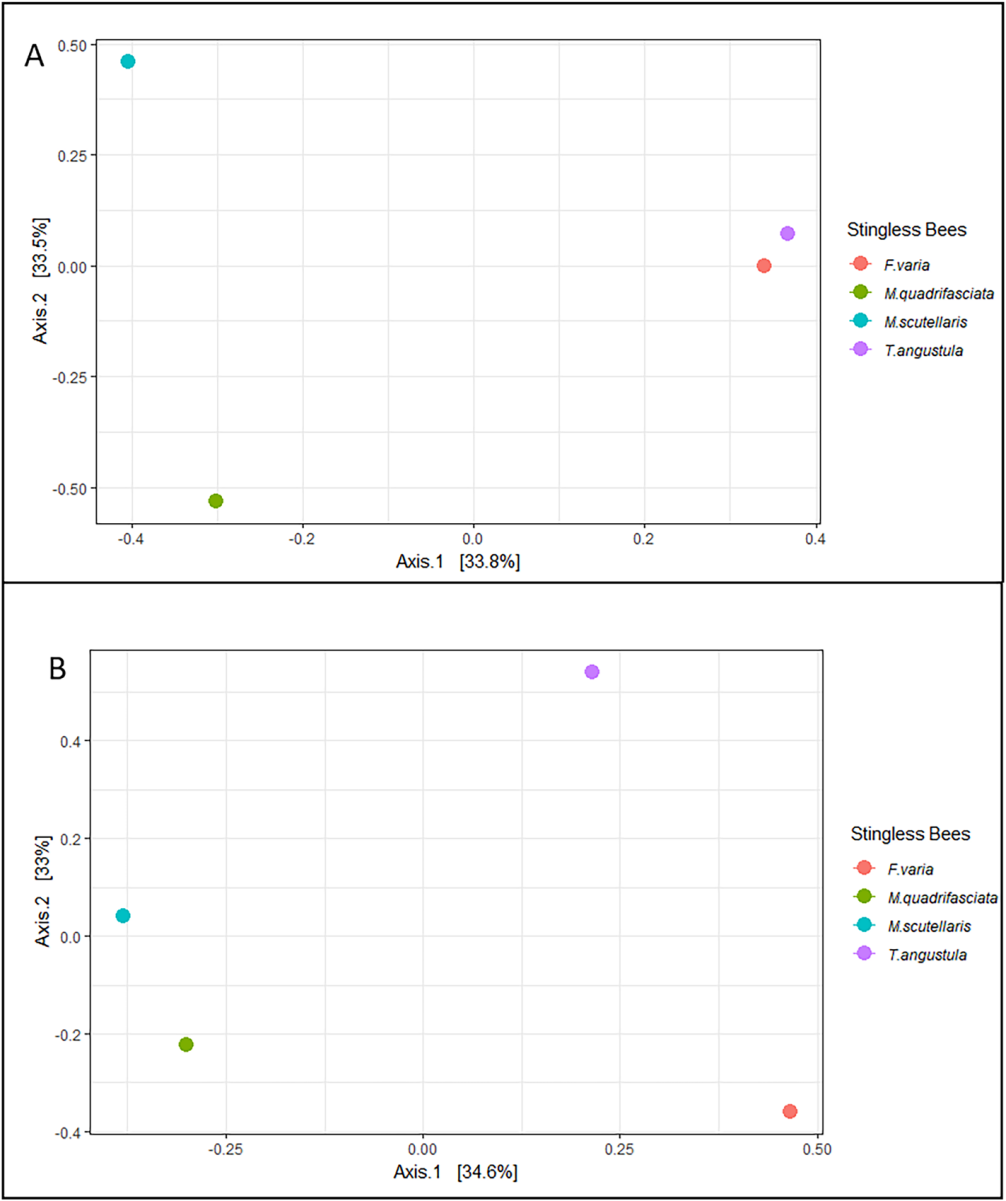
PCoA analysis Bacterial (**A**) and Fungal (**B**) community sequencing.

The Principal Coordinate Analysis (PCoA) of the bacteria community suggests different microbial patterns for *M. quadrifasciata* and *M. scutellaris* compared with *F. varia* and *T. angustula* LF (Fig. 4A). Patterns of *F. varia* and *T. angustula* showed similar compositions and were grouped separately from Melipona stingless bees, suggesting they present a similar species composition. Likewise, the PCoA analysis of the fungal community suggests different microbial among samples (Fig. 4B).

### Cultivable fraction of the microbiome

**Figure 5:**
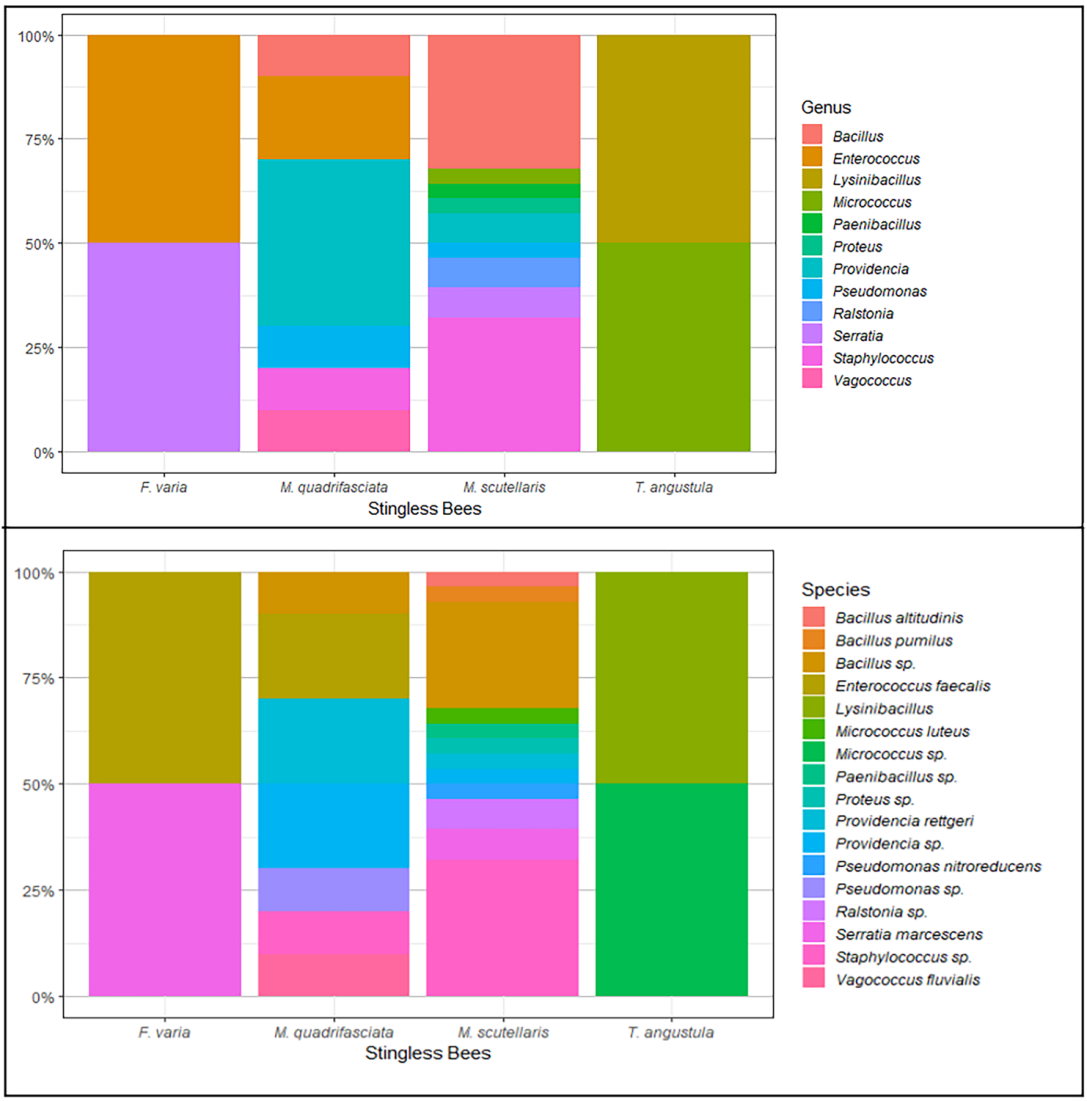
(**A**) Abundance of genera bacterial isolated from larval food of stingless bees. (**B**) An abundance of species was found from the larval food of stingless bees.

A total of 189 bacteria were isolated in culture medium, 41 from LF of *F. varia*, 41 of *M. quadrifasciata*, 71 of *M. scutellaris* and 36 of *T. angustula*. Seventy-five fungi specimens were isolated from LF stingless bees, ten from *F. varia*, 18 from *M. quadrifasciata*, 29 from *M. scutellaris*, and 18 from *T. angustula*. The isolated microorganisms constructed of the Collection of Microorganisms Isolated from Stingless Bee of the Laboratory of Genetics, Institute of Biotechnology at Federal University of Uberlandia (CoMISBee, Table 4). It was possible to identify some of the bacteria by the Matrix Associated Laser Desorption-Ionization—Time of Flight (MALDI-TOF) method, 42 bacteria isolated from the LF of the four stingless bees were grouped into 12 genera (Fig. 5A). The isolated fungi have not yet been identified.

**Table 4:** Number of microorganisms isolated and preserved in the CoMISBee.

Stingless bees	Number of bacteria	Number of fungi
*Frieseomelitta varia*	41	10
*Melipona quadrifasciata*	41	18
*Melipona scutellaris*	71	29
*Tetragonisca angustula*	36	18

Stingless bees of the genus *Melipona* had the highest number of organisms cultivated and identified for MALDI. For *M. scutellaris* were identified 12 bacteria specimens, and eight for *M. quadrifasciata*, *F. varia,* and *T. angustula* had only two bacteria identified (Fig. 5B). The only genera observed by both NGS and culture-based methods were *Enterococcus* and *Serratia,* isolated from *F. varia*, and *Pseudomonas*, isolated from *M. quadrifasciata.*

## Discussion

This work detected bacteria, fungi, and yeasts associated with LF of four species of Brazilian stingless bee (*F. varia*, *M. quadrifasciata*, *M. scutellaris*, and *T. angustula*) present in an urban Meliponary. These microorganisms are essential for colony maintenance, pollen maturation, and honey fermentation of from stingless bees^16,22–24,25^. Due to the lack of data about the microbiome in stingless bee, our goal was to produce the first descriptive data about microbiome present in the LF of these species. As the beehives were reared in the same place and samples were collected simultaneously, the data were suitable to be compared. Differences were found in the LF microbiota of the four stingless bees, showing high variability and richness composition in species^25^.

Sequencing the V3/V4 region of the 16S gene and region ITS1 and inference of ASV allowed assessing the microbial diversity in the LF of the four stingless bee species. The sequencing of the V3/V4 region of the 16S permitted the classification of 116 ASVs into 27 different genera and only five ASVs at the species level. This region was used due to its great taxonomic coverage to genus level^36,37^. This work is the first report of the bacterial species *Acinetobacter junii, Bombella intestine, Corynebacterium lipophiloflavum*, *Serratia symbiotica,* and *Variovorax paradoxus* in stingless bees. The sequencing of 16S regions enabled identifying the microbial community in the LF of stingless bees and the discovery of new species related to stingless bees^38^.

The bacterial community analysis showed that the most abundant bacteria in the LF of the stingless bees investigated were those of the *Lactobacillus* and the family Acetobacteraceae. This abundance has also been previously reported in the honey stomach/anterior intestine of *Melipona*^39,40^. Therefore, itmay explain their presence in food, as the nurser bee transports fermented honey and pollen by ingestion, storage in the honey stomach, and subsequent regurgitation into the brood cells^4,41–43^ .

In this study, 42 bacteria isolated by the culture-dependent method have been identified. No species of the genus *Lactobacillus* were found in these isolates, although we used MRS agar (selective for Lactobacillus—LB 172,234). The absence of these microorganisms in isolation shows that the database used for identification may not have covered the specimens present in CoMISBee, since bacteria of the genera *Bacillu*s and *Lactobacillus* were found using NGS but were not identified and not isolated.

Bacteria of the genus *Lactobacillus* play an essential role in pollen and nectar processing, honey storage, and protection of these bees^24,43,44^. The genus *Bacillus*, although somewhat related to nectar and pollen processing, produces enzymes such as amylase, esters, lipases, proteases, aminopeptidases, phosphatases, and glycosidases^23,45^. Such enzymes had been described as present in the LF of *M. scutellaris*^22^. Also, a significant enrichment for metabolites related to lactose degradation and galactose metabolism showed in the LF of *M. scutellaris*^46^. The presence of this genus in the colonies of *M. quadrifasciata* may be related to the fermentation of pollen and honey and may also protect against pathogens by producing antimicrobial molecules^24,47–49^.

In this investigation, *Paenibacillus sp.* was isolated in *M. scutellaris,* but not identified in the NGS method. Species of this genus are significant producers of antimicrobials and various enzymes^50^. *Paenibacillus polymyxa*, found in the LF of the bee *M. scutellaris*, can produce antimicrobial compounds protecting these bees against pathogens^51^. However, although *Paenibacillus* harbors species beneficial to bees, some species of this genus are disease-causing honeybees^50,52^.

The discovery of bacteria of the genera *Lysinibacillus* and *Serratia* in the colonies of this work may be a warning of the health of these bees. There is only one report of pathogens associated with stingless bees, the bacterium *Lysinibacillus sphaericus* disease-causing in *Tetragonula carbonaria*^53,54^. Moreover, *Serratia marcescens,* an opportunistic disease-causing species in *Apis mellifera*^54,55^ , was found in this investigation in the LF of *F. varia* and *M. scutellaris* through the isolation technique. Specific studies on the impact of these microorganisms on the health conditions of these colonies and the identification of the species *Lysinobacillus sp* using other methodologies are necessary.

Interestingly, the bacteria *Lysinobacillus sp*. and *Serratia marcescens* were not found in DNA sequencing. It might show that other microorganisms present in the LF prevent the growth of *S. marcescens* and keep it so low that the extraction and sequencing technique could not assess this specie. It is essential to highlight that all colonies used for collecting LF were healthy.

The isolation of bacteria in a culture medium is essential for accessing these microorganisms and allowing their biotechnological use. Nonetheless, the fact that it does not identify among the isolated species some bacteria essential for the fermentation of LF reinforces the importance of molecular techniques to elucidate better the microbiome's diversity. Furthermore, this fact emphasizes the importance of using both methodologies to know of the microbiota of stingless bees^38^.

In addition to intimate relationship with bacteria, stingless bees have symbiont relationships with fungi^3,12,14^. The diversity of fungi in the LF of stingless bees was visualized with the sequencing of the ITS1 region. The sequencing of the ITS1 region was effective in showing the fungal composition^56,57^. Distinct species of fungi and yeasts were found in the LF of *F. varia*, *M. quadrifasciata*, *M. scutellaris*, and *T. angustula*. This work reinforces that filamentous fungi and yeasts are closely related to stingless bees ^12–14^.

The diversity of yeast species found in the LF of stingless bees varied among the bee species studied. *Saccharomyces*, *Starmerella,* and *Candida,* are the most common genera in this research. These genera were found in pollen and honey of *F. varia, M. quadrifasciata,* and *T. angustula* stingless bees^58–62^. This group of yeasts is related to pollen and honey fermentation^3,15,60,63^. The yeast *Saccharomyces cerevisiae* was observed in the LF of all bee species in this study. Detecting these organisms in stingless bees paves the way to finding new strains with significant biotechnological potential^58^.The species *Saccharomyces cerevisiae*, *Starmerella meliponinorum, and Rhodotorula mucilaginosa* were detected in *T. angustula*, and our work, suggesting that these yeasts may be related to the hives of these stingless bee species.

The highest diversity of yeast species was observed in the LF of *M. quadrifasciata*, including the genera *Diutina*, *Meyerozyma*, *Rhodotorula,* and *Starmera*, found only in the LF of this stingless bee. The genus *Bannoa* found only in *M. scutellaris*, the first report of the species *Bannoa ogasawarensis* related to a stingless bee. This specie has been reported to colonize dead leaves of plants^64^. The osmophilic yeast *Zygosaccharomyces mellis,* found only in *T. angustula* LF, was reported in the honey of the *T. angustula*^65^. The *Zygosaccharomyces* genus was described to be involved in food deterioration in the hive, but it can also establish mutualistic relationships with other organisms^3,66^.

This work observed filament fungi in the LF of all stingless bee species. *Cladosporium* was the only genus common among the four species^67–69^. The *Cladosporium* was previously reported in stingless bee of the *Melipona* genus^69^. The most representative filamentous fungi in the stingless bees species are described as phytopathogens^70,71^. However, they are used as a biological control for various pathogens. *Epicoccum dendrobii* produces biomolecules capable of inhibiting phytopathogens^72^. Twenty-one species of *Penicillium* and 16 of *Talaromyces* were observed in *M. scutellaris*^75^. The genus *Penicillium* were detected in the LF of *F. varia* and *M. quadrisfaciata* and *Talaromyces* in *M. quadrifasciata*.

Stingless bees may use spores produced by fungi as a protein source in times of pollen scarcity since the nutritional value of the spores is lower than that of the pollen^21,74,75^*. T. angustula* presented the most remarkable diversity of filamentous fungi and is considered a specialist species in colonizing diverse niches. Therefore, it can use fungi as a protein source for to maintain the colonies^60^.

The diversity of bacteria and fungi in stingless bees follows the diversity indexes evaluated. On the one hand, bee species with a more extraordinary richness of bacteria present a lower richness of fungal species, as occurred in *F. varia*. On the other hand, the inverse relationship between bacterial and fungal species richness is genuine, as occurred *in T. angustula*. The yeast diversity found in the LF in our work is like the yeast diversity observed in the honey of the species *F. varia, M. quadrifasciata,* and *T. angustula*^61^, indicating the diversity of yeasts is related to the honey of these species.

In addition, fungi and bacteria are organisms known for their ability to produce metabolites with antimicrobial activity. Bacteria of the genera *Bacillus*, *Lactobacillus*, *Micrococcus*, *Pseudomonas*, *Providencia*, *Serratia*, and *Vagococcus,* found in this work, can produce compounds that suppress the growth of other bacterial genera^76–79^. Fungi of the genera Cladosporium, Aspergillus, or Penicillium can reduce the number of bacteria in LF by secreting antimicrobial molecules capable of inhibiting bacterial strains^67,68,80,81^. This fact may explain the difference in the composition of fungi, yeasts, and bacteria in the LF of the different bee species.

Our findings on the microbiota of LF reinforce the importance of vertical transmission of the microbiota from social contact (trophallaxis) between individuals in colonies^82^. Furthermore, they suggest that part of the microbiota associated with LF can pass between bees in the nectar collection process and during the transfer of honey and matured pollen to the brood cells^40,43^.

This work opens a new perspective in the knowledge of Brazilian stingless bees' microbiomes, which still needs to be explored. In addition, the microorganisms found here can be an essential source for discovering and prospecting novel bioactive compounds. Recently, our group showed that some of these isolated bacteria (genera *Providencia, Serratia,* and *Vagococcus*) have a biotechnological potential, showing antimicrobial activity against multiresistant hospital bacteria^76^.

This research described the microbiota associated with the species *F. varia*, *M. quadrifasciata*, *M. scutellaris,* and *T. angustula* and discussed their possible roles in maintaining and protecting of the colonies. This work is the first investigation about the microbiota of the LF of stingless bees. It opens the pathway for elucidating the role of bacteria and fungi in the survival of these organisms. Besides that, the first biobank with 189 bacteria and 75 fungus isolates from the LF of Brazilian stingless bees was also created. It can be used for different studies, such as the screening of bioactive compounds with the potential for treating a wide range of diseases.

## Methods

### Sample collection

**Figure 6:**
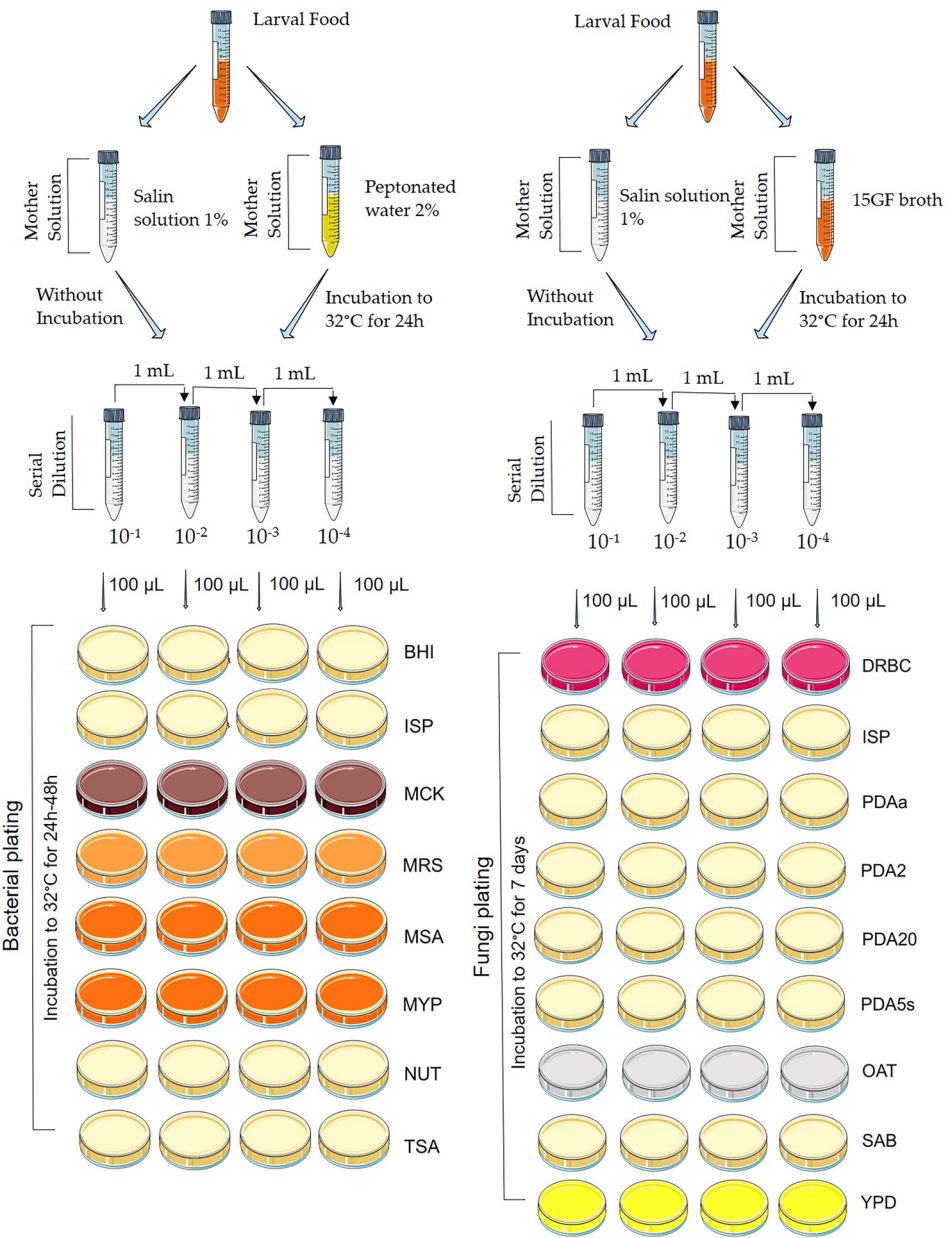
Methodology for cultivating larval food from four stingless bee species with different dilutions and culture mediums. Brain Heart Infusion (BHI), Nutrients (NUT), Tryptone soy agar (TSA), Man, Rogosa and Sharpe (MRS), Mac Conkey (MCK), Mannitol Salgado (MAS), Yeast Malt Agar (ISP), Potato Dextrose Agar 2% (PDA2) and 20% (PDA20) glucose, PDA acidified with tartaric acid (PDAa), PDA NaCl 5% (PDA5s), Oatmeal Agar (OAT), Yeast Extract-Peptone-Dextrose (YPD), DRBC (Dichloran Rose-Bengal Chloramphenicol) e Sabouraud (SAB). *Images of representative method from ‘Smart Servier Medical Art’ *(https://smart.servier.com/).

The LF samples were collected from only one beehive of each of the four stingless bees’ species (*Frieseomelitta varia*, *Melipona quadrifasciata*, *Melipona scutellaris,* and *Tetragonisca angustula*) and processed to obtain microorganism isolated by the culture technique (Fig. 6) or for DNA extraction. The beehives were kept in an urban meliponary in Uberlândia city, Minas Gerais, Brazil. All samples were collected in the springer season, October 2018.

### Larval food collection

Brood cells were collected from beehives and stored in sterile Petri dishes. In the laminar flow cabinet, the brood cell was cleaned with sterilized distilled water for 1 min and rinsed three times with 70% ethanol. Then, the brood cells were opened with a sterile pipette tip, the eggs were removed, and the LF was collected (the LF from the cell with larvae was discarded). At least 1.0 mL of LF was collected from the brood cells of each colony.

### Molecular characterization of the microbiome

#### DNA extraction

One hundred microliters of LF collected were used for DNA extraction, following the manufacturer’s instructions for bacterial and fungal DNA extractions using the DNeasy® Blood & Tissue (Qiagen) kit. The DNA quantity was measured using NanoDrop 2000 Spectrophotometer, and quality was analyzed by agarose gel 1% electrophoresis stained with ethidium bromide.

#### Amplicon sequencing and data analysis

For the construction of libraries, 30 ng of DNA was used as input to amplify the V3/V4 region of the 16S rRNA gene with primers 341F (CCTACGGGRSGCAGCAG) and 806R (GGACTACHVGGGTWTCTAAT)^36,37^, or ITS1 region with the primers IST1(GAACCWGCGGARGGATCA) and ITS2 (GCTGCGTTCTTCATCGATGC)^56,83^. The amplicons were sequenced in the paired-end reads (2 × 250 bp) of 500 cycles on the Illumina MiSeq platform. The raw data was processed using the package DADA2 in R version 4.0.2^84^ to generate the Amplicon Sequence Variants.

The reads of the V3/V4 region of the 16S rRNA gene were analyzed according to the “DADA2 Pipeline Tutorial” (https://benjjneb.github.io/dada2/tutorial.html). The workflow has been changed in the *trinLeft* parameter for trimming the primers and using the values 17 for forward, 20 for reverse reads, and *truncLen*; the forward reads were truncated to 290 and reverse reads to 200 sizes. Taxonomy was assigned with Silva 138 database^85^.

The amplicons of the ITS1 region were analyzed according to “DADA2 ITS Pipeline Tutorial (1.8)” (https://benjjneb.github.io/dada2/ITS_workflow.html). The Cutadapt^86^ was used to remove the primers and the *minLen* parameter to adjust the sequencing quality. The workflow has been changed in parameters *trimLeft* (default 0), *truncLen* (default 0), and *minLen*, removing reads with lengths less than 50. The Amplicon Sequence Variants (ASV) were classified using UNITE ITS database using pre-trained.

The alfa and beta diversity analyses were performed with phyloseq, ggplot2, and vegan packages in R^87^ using the “DADA2 Pipeline Tutorial”.

Microbiome analysis by cultivation-based techniques and construction of biobank.

#### Microorganisms’ isolation

Three hundred microliter of LF were collected from brood cells of each colony and used to prepare of three different solutions. First, one hundred microliters of the sample were resuspended in 10 ml of solution 1% NaCl, 100 μL of LF in 10 ml of peptone water, and 100 of LF μL in 10 ml of 15GF broth^26^, as shown in Fig. 1^25^. Then, the second and third solutions were incubated for 24 h at 32ºC ± 0,5 (Incoterm® Dual Sensor Digital Thermo-Hygrometer) because of is the average internal hive temperature.

All these three solutions were named “mother solution”. From the mother solutions, the serial solutions were made to the order of 10^–4^, and 100 μL of each was plated on the surface of different culture mediums and incubated in a bacteriological incubator at 32ºC ± 0,5 and checked for 48 h for bacterial growth and seven days for fungal growth.

After the incubation, each colony was isolated on BHI agar for bacteria and PDA agar for fungi. Colonies were characterized by visible morphological differences and gram staining (Newprov). The isolated microorganisms were preserved in LB broth (Luria Bertani) plus 20% glycerol and kept in an ultra-freezer at −80ºC.

#### Identification by MALDI-TOF of cultivable bacteria

The bacteria were plated on BHI for taxonomic identification and incubated at 37ºC ± 1 for 24 h. An isolated colony of each strain was collected from the agar using an inoculation loop and inactivated with absolute ethanol. Bacterial preparation was performed following the manufacturer's protocol^88^. Spectra were acquired using a Flex Control Microflex LT mass spectrometer (Bruker Daltonics) with a 60 Hz nitrogen laser. The spectra of each sample were processed using MALDI Biotyper software version 3 (Bruker Daltonics) and assembled to generate a Master Spectral Library (MSP) for strains using the BioTyper MSP breeding standard^88,89^.

## Supplementary Information

Supplementary Information 1.

Supplementary Information 2.

## Data Availability

The files have been deposited in the SRA (Bioproject: PRJNA860336).
